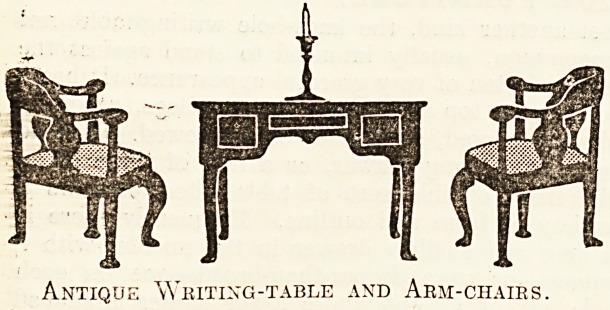# Collecting Antique Furniture

**Published:** 1908-12-26

**Authors:** W. Heneage Legge


### THE PRACTITIONER'S RELAXATIONS.

### COLLECTING ANTIQUE FURNITURE.

WE have already dealt with the subject of collecting old oak chests, coffers, and kindred articles, carved, panelled, or plain, as a hobby or recreative pursuit suitable for a doctor to adopt. There are, however, many other forms of antique furniture well worth the attention of the collector, both from the point of view of their use and beauty, their value, and their interest as specimens of man's handicraft.

Such are antique chairs, tables, cupboards, benches, and stools. The former are those that present the greatest variety in general design and in detail; they are also perhaps those to which the greatest money value attaches, particularly those of the Chippendale, Sheraton, or Hepplewhite make. So much publicity is given to the subject that it is needless to enlarge on the high prices given for fine specimens of Chippendale chairs, and such are usually beyond the purse of the practitioner. Nevertheless there are yet to be picked up old mahogany, rosewood, or walnut chairs of the desirable period and style; some of them more beautiful in line and curve though less elaborate in design than those which command such high prices. Though often bearing evidence of rough treatment or unskilful attempts at repair, their original make was so solid and sound that they outlast generations of chairs of modern manufacture. Not unfrequently antique bedside chair-commodes were made in the aforesaid styles, and may be met with occasionally in cottages and farmhouses. Most of them are quite adaptable, with a little ingenious alteration, to more ornamental uses. I possess two such articles whose promotion is in the one case quite unlikely, in the other impossible of detection. Chairs of an older period are scarcer, though they may be met with now and again. Upon one occasion I purchased a fine old high-backed Jacobean chair immediately after a dealer had, as he thought, cleared out all "the old stuff" from the dwelling of a deceased cottager. It had escaped notice, owing to being covered from top to bottom in cretonne, from carved straight back to carved curved legs.

There are still about the countryside considerable numbers of oak and ash rush-bottomed chairs, chiefly of the "ladder-back" description, which, if not very ancient and rather plain, are decidedly not modern, and are at once pleasing in appearance and strong in make.

Antique tables present the collector with a great variety of make in shape, size, and design. One of the most common is that called "eight-legged." Nowadays they are often called "gate-legged," possibly by corruption or misunderstanding of the former word, albeit the name has a certain applicability. They are generally of moderate size, but occasionally very large ones are to be seen. Of the other extreme, which is rare, I possess a very pretty little specimen.

Another description of antique table often met with is the card-table, round, oval, or rectangular. Yet another kind, the knee-hole writing-table, not uncommon, usually intended to stand against the wall, is often of very graceful appearance. It has a rectangular top and slender tapering legs, generally slightly curved, sometimes boldly bowed. Instead of a single long drawer, or a row of three alike, the front of this sort of table often presents a variety of form and outline. Frequently there is a long and shallow drawer in the middle, with a square—or even deeper-than-broad—one on each side, set in a front whose lower border is shaped into a pleasing outline. All this, with pretty brass handles and keyplates, and perhaps the further elaboration of inlay, forms a highly-ornamental as well as very useful piece of furniture.

HEPPLEWHITE CHAIR.

As for antique chests of drawers they offer a great field of choice to the collector, so varied are they in form, size, design, and ornamentation. Some show beauty in outline, some in the grain and colour of their wood, some in the pattern of their inlay. A large proportion have suffered at the hands of our early Victorian forbears the ruthless removal of their brass drop-handles or elaborately-pierced handle and key-plates, hideous china or wooden knobs having been substituted. It is, however, often possible to replace these substitutes by other old handles of similar design to the original, or to make copies of the old brasses. The latter is made practicable when the outlines of the ancient plates are visible, as is often the case, beneath the coatings of more modern varnishings. Though this is a laborious work and by no means easy for an amateur, it is an effort after truth and beauty that is well repaid in the resulting restoration. The eighteenth-century "tall-boy" chests of drawers are very desirable acquisitions, being extremely useful in their capacious proportions and usually much too ornamental in line and detail to be allowed to remain in bedrooms—their original destination. Sometimes they have short bow legs; more often

Antique Writing-table and Arm-chairs.

a pleasingly-shaped plinth, while the front is in some cases elaborately inlaid, in others panelled, while the brass handles, if remaining, show considerable beauty and variety. Often, too, they were veneered as well as inlaid, and unless they have been more than usually well-cared for this wooden epidermis is generally found to be considerably denuded in conspicuous places and very much in need of repair. Such cases are best treated by calling in the services of a professional cabinet-maker, a somewhat costly proceeding, but the most satisfactory one in the end.

Kindred in nature to chests of drawers are those useful articles of antique furniture, bureaus. Very varied in material and size, their essentials also differ much in design and construction, particularly in such matters as secret drawers and other internal arrangements; matters, however, which considerations of space will not at present permit of being described. W. Heneage Legge.

## Figures and Tables

**Figure f1:**
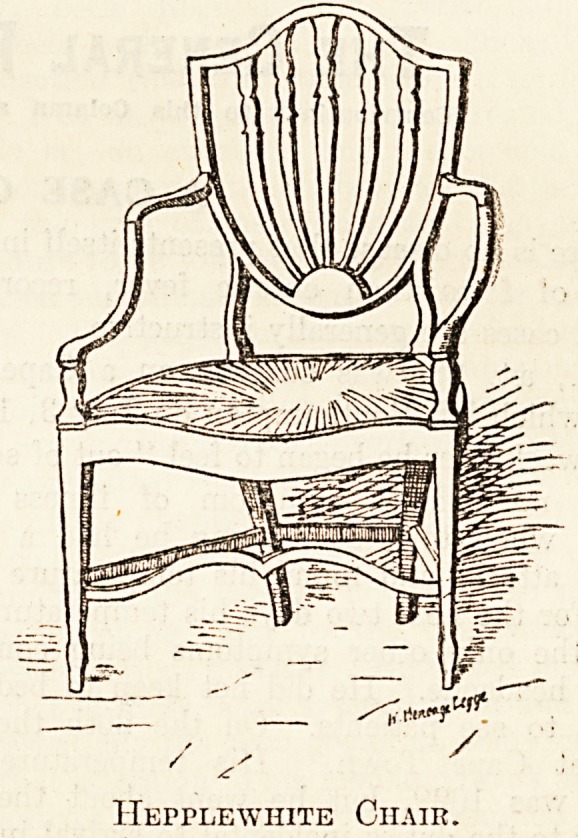


**Figure f2:**